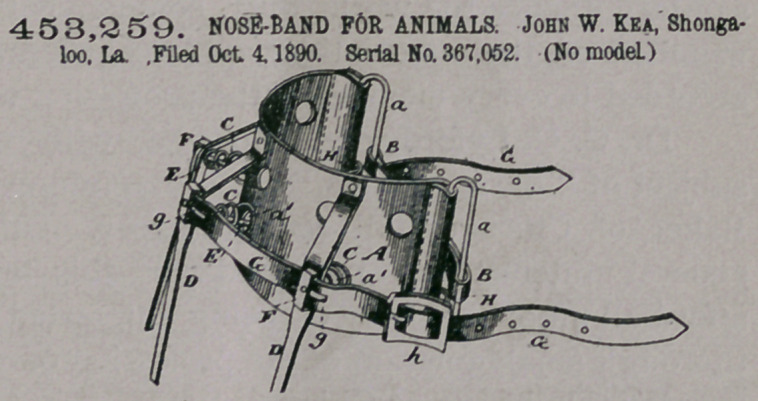# Recent Patents

**Published:** 1891-07

**Authors:** 


### RECENT PATENTS

RELATING TO

### VETERINARY MEDICINE AND ANIMAL INDUSTRY.

---

*Issued by U. S. Patent Office for Month ending June,* 1891.

---

**453,718. NOSE-BAG.** GEORGE D. LEONARD, New Haven, Conn. Filed May 14, 1890. Serial No. 351,802. (No model.)

*Claim.*—1. The combination, with a nose-bag having pulleys at its upper edge, of a cord forming a loop over the head of the animal, extending down through the pulleys on the bag and back through slide or buckle S, secured to a hook at the back of the animal, and a separate cord extending from said buckle over the top of the animal's head to the bag, substantially as described.

2. The combination, with the nose-bag having loops, as described, of a cord forming a loop at the top of the head of the animal, extending down through the pulleys of the bag and back through slide or buckle S, which is connected to the back hook, a halter or bridle having loops which embrace said cord at the sides and top of the head of the animal, and a cord extending from the bag and loop at the top of the head through slide S, substantially as described

**453,804. WATERING DEVICE FOR STOCK-CARS.** SIEGFRIED M. FISCHER, Chicago, Ill. Filed Dec. 13, 1890. Serial No. 374,633. (No model.)

*Claim.*—1. The combination, with a stock-car, of the elevated water-pipe E, arranged at the roof portion of the car and provided with depending branches F, extended down inside the car and bent laterally to the outside or front thereof, the rotating troughs C, and short pipes *g*, supporting the troughs and connected with the lower entremities of the said depending branches, substantially as described.

2. The combination, in a stock-car, of the fixed frame A, the elevated water-pipe E, having depending branches F, provided with short pipes *g*, the rotating troughs C, supported by the short pipes, the swinging false side B, hinged at its lower portion, and direct-acting link-bars *c*, pivotally connected to the rotating troughs and to the swinging false side, substantially as described.

3. The combination, with a stock-car having the fixed frame A and swinging false side B, hinged at its lower portion, of the elevated water-pipe E, arranged at the roof portion of the car and having depending branches F, extending downward inside the car and bent laterally through the fixed frame to the outside or front thereof, the rotating troughs C, the short pipes *g*, supporting the troughs and connected with the said depending branches, and the link-bars *c*, pivotally connected to the troughs and to the swinging false side, substantially as described.

**453,414. BRIDLE-BIT.** JOEL MCKENNEY, Chelsea, Mass. Filed Mar. 9, 1891. Serial No. 384,236. (No model.)

*Claim.*—The herein-described snaffle or bridle bit, consisting of two bars, the inner ends of which meet or nearly meet, said bars being joined together by means of a link connected therewith at an appreciable distance beyond the meeting ends, the inner portions of the bars being slotted, and a ring *e*, arranged in the said slots at a right angle to the said link, as set forth.

**453,675. DEHORNING IMPLEMENT.** THOMAS W. SPRAGUE, Minneapolis, Minn. Filed Jan. 12, 1891. Serial No. 377,409. (No model.)

*Claim.*—The combination, in a device of the class described of a long pole-handle 2 and curved lever pivotally connected with said handle and provided with the convex knife 8, detachably secured to the end of said lever at one side thereof, a ring 12, arranged to slide upon the pole 2, the curved lever 10, secured to said ring and pivotally connected to the lever 7 at 9, the concave knife 11, detachably secured to one side of the lever 10 opposite said knife 8, and the rod 13, secured to the ring 12 and provided with a handle 15 at its end, all substantially as described.

**453,943. BRIDLE.** HENRY SCHMITZ, Wymore, Nebr. Filed Jan. 26, 1891. Serial No. 379,111. (No model.)

*Claim.*—1. The bridle having its side or cheek straps each passed up and over the top of the head-piece, crossing each other thereat, passed through an anti-friction loop, and terminating each on the opposite side of the bridle in a loop or eye, substantially as specified.

2. In a bridle, the combination with the head, throat, and brow-straps, the bit, and their connections, of the side or cheek straps connected at one end to the bit-rings and passed up through loops over the head-piece, crossing each other at the central top portion of said head-piece, passed through a loop thereon, thence extended down on opposite sides of the bridle, and terminating each in a loop for the bridle-rein, substantially as specified.

3. In a bridle, the combination, with he cheek or side straps, each passed up and through a double roller-loop at the top of the head-piece, thence respectively extended down the opposite sides of the bridle and terminating each in a loop or eye, of the bridle-reins connected to the bit and passed through said loops or eyes and having a connection with the reins, substantially as specified.

**454,210. RUBBER PAD FOR INTERFERING-STRAPS.** MICHAEL HAUGHEY, St. Louis, Mo. Filed Dec. 29, 1890. Serial No. 376,015. (No model.)

*Claim.* – The improved article of manufacture comprising the strap and buckle, the rubber loop-pad in the rear of the buckle and adjustable on the strap, and the adjustable pad C, having loop *c*1 and plate *b*, secured in said loop and carrying the pendant, all substantially as shown and described, and for the purposes specified.

**453,719. NOSE-BAG.** GEORGE D. LEONARD, New Haven, Conn. Filed May 23, 1890. Serial No. 352,868. (No model.)

*Claim.*—1. A nose-bag body consisting of a tube, of canvas or similar flexible material, having a hem or distending-piece at its lower edge, in combination with a bottom of rigid material having a rabbet at its lower corner, into which the corded edge of the body extends, substantially as described.

2. The combination, with a tubular bag-body having a hem at its lower edge, and a cord inclosed therein, of a wooden bottom rabbetted at the lower corner, so that the corded canvas lies in the rabbet, and securing means for holding the bottom into the bag, substantially as described.

**453,830. HITCHING DEVICE FOR ANIMALS.** ALFRED DAUPHINAIS, Hurley, Wis., assignor of one-half to Alexandre Lalond, same place. Filed Dec. 22, 1890. Serial No. 375,484. (No model.)

*Claim.*—1. In a hitching device for animals, the combination of an appertured block provided with a seat at one end of the apperture therein and a clamping-pin having one extremity thereof pivoted to the block in line with said seat, substantially as set forth.

2. In a hitching device for animals, the combination of an apertured block provided with a seat at one end of the aperture therein, a bracket at one side of the block and in line with said seat, and a clamping-pin having one extremity thereof pivoted to the bracket, substantially as set forth.

3. In a hitching device for animals, the combination of an apertured block provided with a seat transverse to the aperture therein, a bracket on one side of said block, and a clamping-pin pivoted to said bracket and having the central portion thereof reduced in diameter and polygonal in form, substantially as set forth.

**453,197. ANIMAL-POKE.** WILLIAM P. MARQUIS, Marquette, Nebr. Filed Feb. 20, 1891. Serial No. 382,190. (No model.)

*Claim.*—1. An animal-poke comprising the plate or bar designed to be secured to the head of an animal, the barb-lever fulcrumed at the outer end of the bar or plate and arranged to engage the nose of an animal, and the operating-lever connected with the barb-lever and arranged to be depressed to force the barb-lever into the nose of an animal, substantially as described.

2. An animal-poke comprising a plate or bar designed to be secured to the head of an animal, the barb-lever fulcrumed at the outer end of the bar or plate and arranged to engage the nose of an animal, the operating-lever fulcrumed intermediate its ends and arranged at an angle to the plate or bar and having one end connected to the barb-lever, and the spring interposed between the operating-lever and the plate or bar, substantially as described.

3. In an animal-poke, the combination of the plate or bar provided with fulcrum-posts 2 and 6, the barb-lever mounted on the post 2, the operating-lever fulcrumed on the post 6 and connected to the barb-lever, the rod secured to the plate or bar and provided with a longitudinal slot to receive the operating-lever, and the spring arranged on the rod and interposed between the operating-lever and the plate or bar, substantially as described.

4. In an animal-poke, the combination of the plate or bar provided at its inner end with the longitudinal slot 11, the barb lever arranged at the outer ends of the plate or bar, the operating-lever connected to the barb-lever, the strap 19, arranged at the outer end of the lever, the extention 12, adapted to be adjusted and provided with a set-screw arranged in the slot 11, and the strap or band 17, substantially as described.

**453,508. VETERINARY INSTRUMENT.** JOSEPH RUBY, Beckleysville, Md. Filed Mar. 18, 1891. Serial No. 385,527. (No model.)

*Claim.*—1. A veterinary instrument comprising the pistol, the plunger arranged within the barrel, the rod connecting the plunger and the hammer, the curved arms arranged at the end of the barrel and the elastic piece 15, centrally secured to the plunger and having its ends connected to the arms, substantially as described.

2. A veterinary instrument comprising the pistol having its barrel provided with the shoulder 5, the spiral spring arranged within the barrel and having its inner end bearing against the shoulder, the plunger arranged within the barrel and being engaged with the outer end of the spring, the rod connecting the plunger and the hammer, and the spring-arms secured to the outer end of the barrel and adapted to hold medicine, substantially as described.

3. In a veterinary instrument, the combination of the pistol, the screw 20, arranged at the butt of the pistol, and the metal block provided with a recess to receive the rear end of the mainspring and secured to the screw, substantialy as described.

3. In a veterinary instrument, the combination of the pistol, the screw 20, arranged at the but of the pistols, and the metal block provided with a recess to receive the rear end of the mainspring and secured to the screw, substsntially as described.

4. The barrel and the plunger therin, combined with the elastic piece 15, centrally secured to the plunger, the curved spring-arms 16, to which the ends of the elastic piece are secured, and the springs 17, attached to the barrel and connected to the ends of the arms 16, substantially as described.

5. The barrel and the plunger therein, combined with the elastic piece 15, centrally secured to the plunger, and the curved spring-arms 16, said arms being composed of plates of sheet metal which are flexibly connected to the barrel, substantially as described.

**453,940. NOSE-RING FOR ANIMALS.** EDMUND K. REA, Ovid, Mo. Filed Sept. 1, 1890. Serial No. 363,649. (No model.)

*Claim.*—A nose-ring consisting of two hinged sections, one of the said sections having a slot in the outer side of its free end, the end of the said slot being beveled and the opposite section having its free end beveled from its inner face to circumference and being reduced in thickness, forming a tongue, the base of the said tongue being beveled outward toward the sides, and a pin passing through the walls of the said slot and through the body of the said tongue, as described.

**454,436. VETERINARY FILE.** FRANK K. HESSE, Boston, Mass., assignor to Codman & Shurtleff, same place. Filed Jan. 17, 1891. Serial No. 378,064. (No model.)

*Claim.*—1. In a horse tooth-file, the handle and shank or equivalent, the base *b*, having the lip at its outer end, and vertical lips, as *b*1, at each side to receive and hold the file, combined with a removable locking device at the heel end of the file, consisting of a plate of suitable width to enter between and be guided by said vertical lips *b*1, substantially as described.

2. In a horse tooth-file, the handle and shank or equivalent, and the base *b*, having the guideway *e*, combined with the file, the inturned lip *b*2 at one end, and the locking device *d*1 at the other end, having the projection *e*1 following in said guideway, substantially as described.

3. In a horse tooth-file, the handle and shank or equivalent, and the base *b*, combined with the file beveled at its ends, the inturned lip *b*2 at one end, and the removable locking device *d*1 at the other end, substantially as described.

4. In a horse tooth-file, the file and holder for it and the locking device for said file, combined with a two-part shank *a*1 *a*4, and a screw-threaded screw-driver socket in the portion *a*4, substantially as described.

**453,932. ANTI-CRIB-BITING DEVICE FOR HORSES.** ANTHONY LYNCH, Philadelphia, Pa. Filed Mar. 11, 1891. Serial No. 384,583. (No model.)

*Claim.*—1. A device for preventing an animal from crib-biting, said device having a tongue projecting into the mouth of the animal between the teeth of the upper and lower jaws, substantially as described.

2. The combination of a band extending around the nose of the horse and into the mouth of the rear, with a band extending over the nose between the nostrils and into the front of the mouth between the front teeth and attached to the rear band at the back of the mouth, substantially as described.

3. The combination of the rear band with the front band B, attached to the rear band A at its ends, and a plate on the front band in line with the front teeth when the device is applied to the mouth of the animal, substantially as described.

4. The combination of the rear band and the front band, both extending into the mouth of the horse, straps by which the device is secured to the halter, and a strap passing under the lower jaw of the animal, substantially as described.

5. The combination of the rear band, the front band, devices for securing the bands to the halter, and plates on each side of the front band and in line with the front teeth of the animal, substantially as specified.

6. The combination of the front and rear bands secured together, with plates on each side of the front band in line with the teeth of the horse, and a tongue-piece below the band, substantially as described.

7. The combination of the front and rear bands, the straps for securing the bands to the harness, with filling-pieces for increasing the thickness of the front band at the point where it crosses the line of the front teeth of the horse, so as to open or close the mouth more or less, substantially as described.

**453,817. REGULATOR FOR FEED-BOXES.** JOSEPH S. MILLS, Union, Ill. Filed Jan. 29, 1891. Serial No. 379,514. (No model.)

*Claim.*—1. A feed-regulator for horse feed-boxes, consisting of a receptacle having a contracted lower end, a flexible front, and rigid sides, said front portion secured at its edges to the sides.

2. A feed-regulator for horse feed-boxes, consisting of a receptacle having a contracted lower end, a scoop tapering to the forward end, the height and taper of the sides of said scoop being such as to limit the distance it may extend into the feed-receptacle.

**453,259. NOSE-BAND FOR ANIMALS.** JOHN W. KEA, Shongaloo, La. Filed Oct. 4, 1890. Serial No. 367,052. (No model.)

*Claim.*—The fingers D, having outside keepers F, strap G, passing through said keepers and fastened thereto and to the fingers, the straps H H, and the apertured nose-strap A, having end loops connected by a buckle-strap B, all combined with prickers E on the under side of fingers D and surrounded by spiral springs, whereby the device may be applied to the nose of an animal, as and for the purpose described.

## Figures and Tables

**Figure f1:**
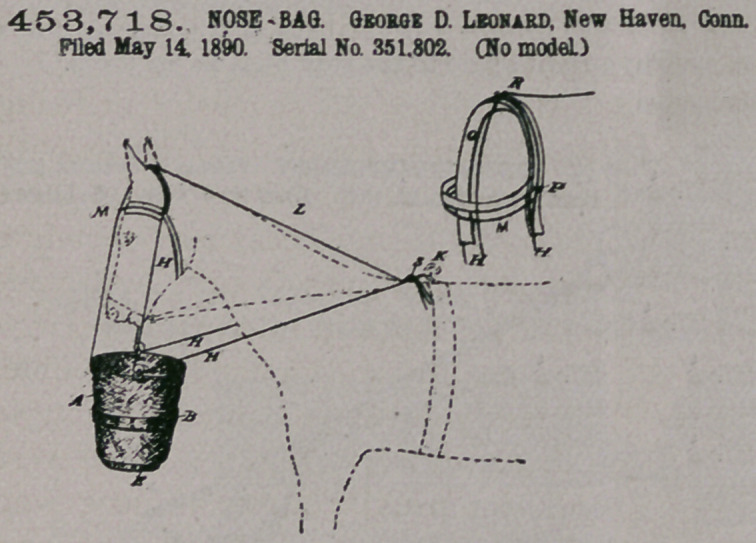


**Figure f2:**
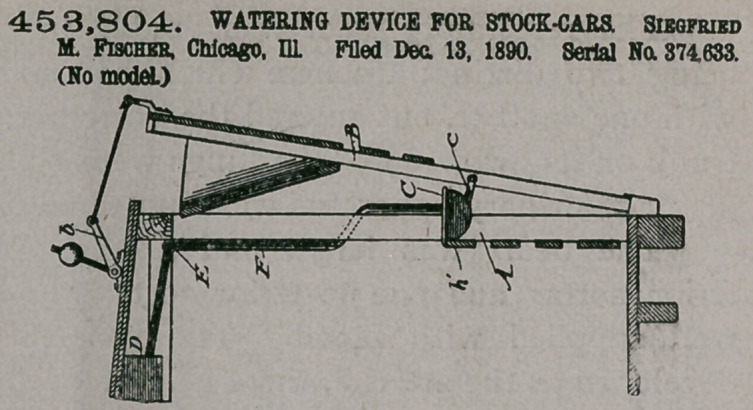


**Figure f3:**
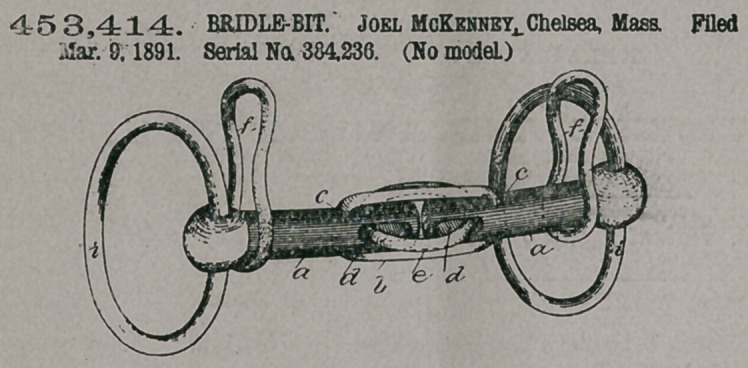


**Figure f4:**
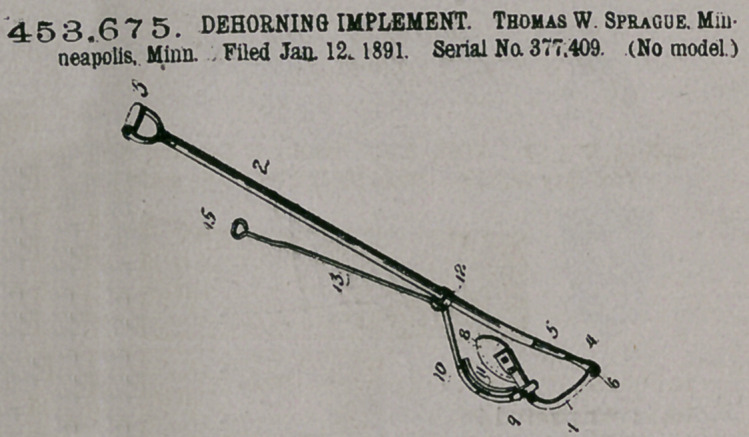


**Figure f5:**
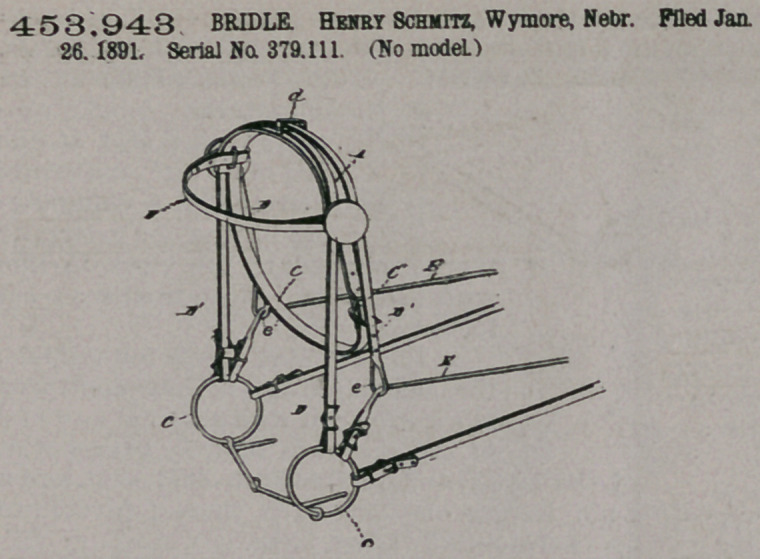


**Figure f6:**
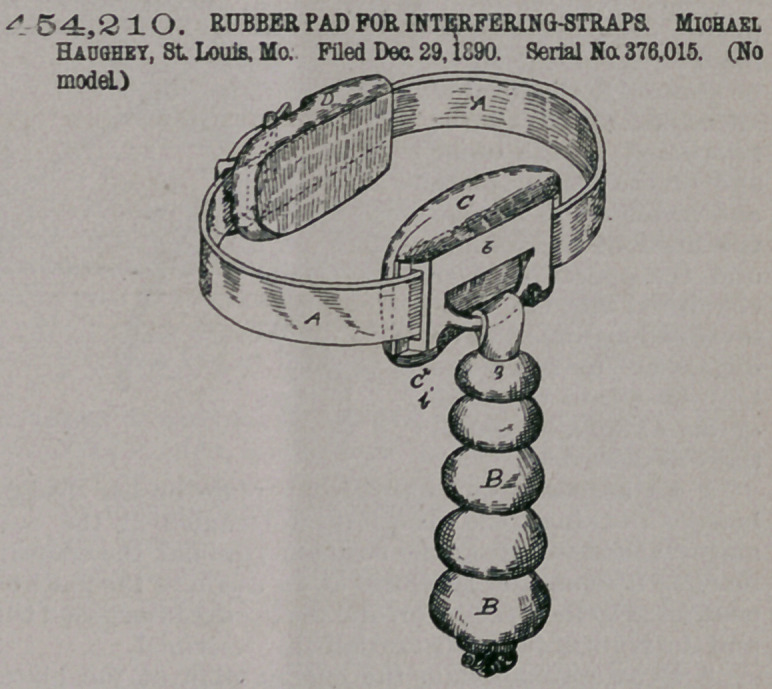


**Figure f7:**
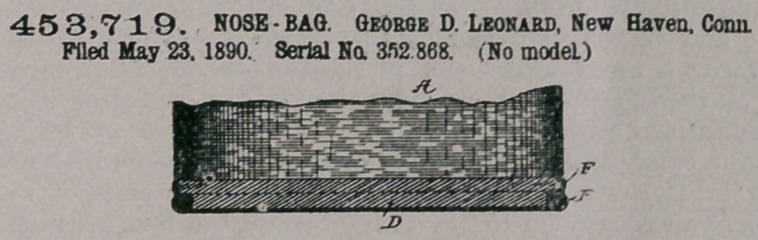


**Figure f8:**
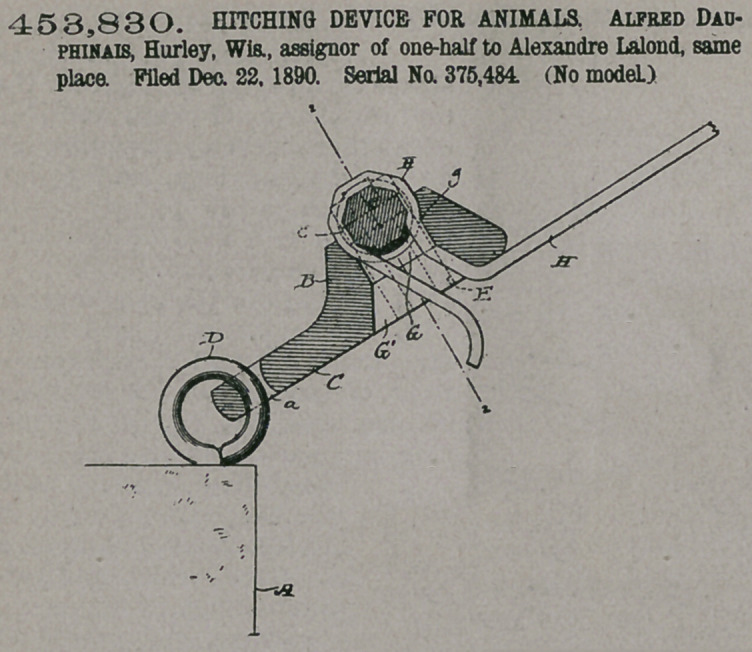


**Figure f9:**
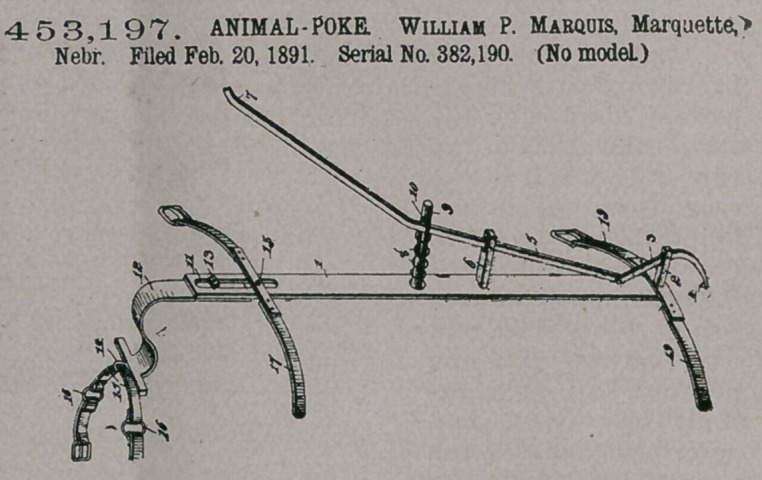


**Figure f10:**
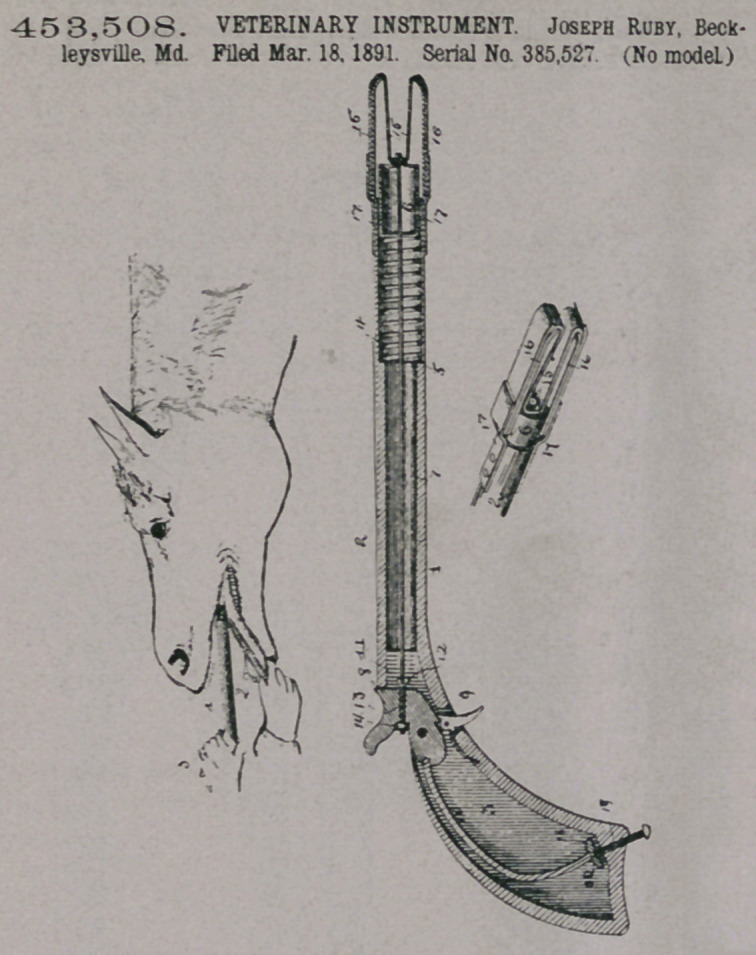


**Figure f11:**
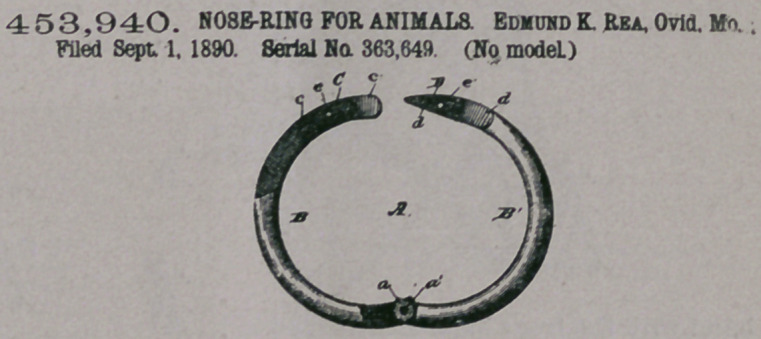


**Figure f12:**
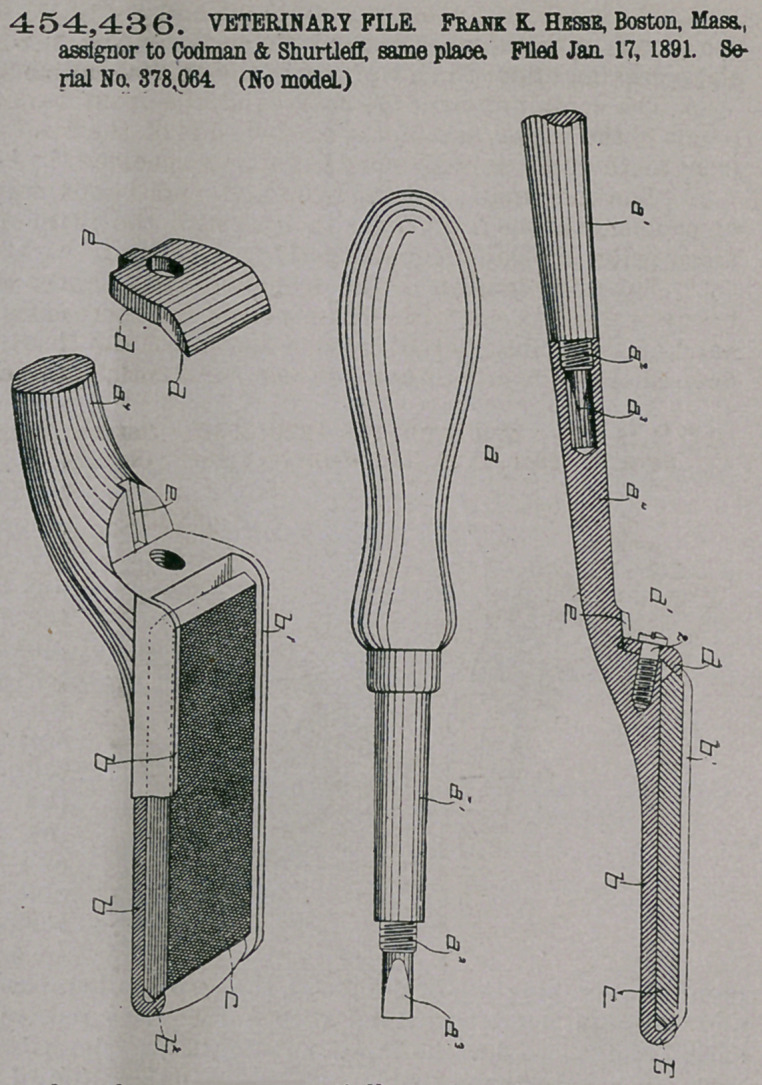


**Figure f13:**
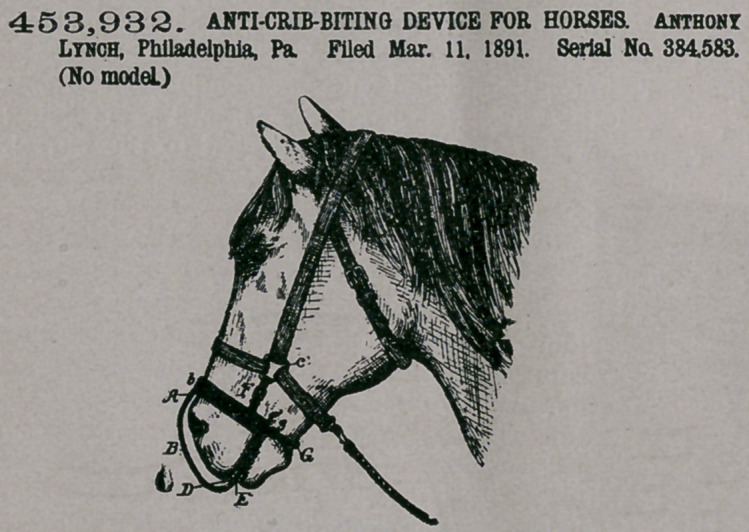


**Figure f14:**
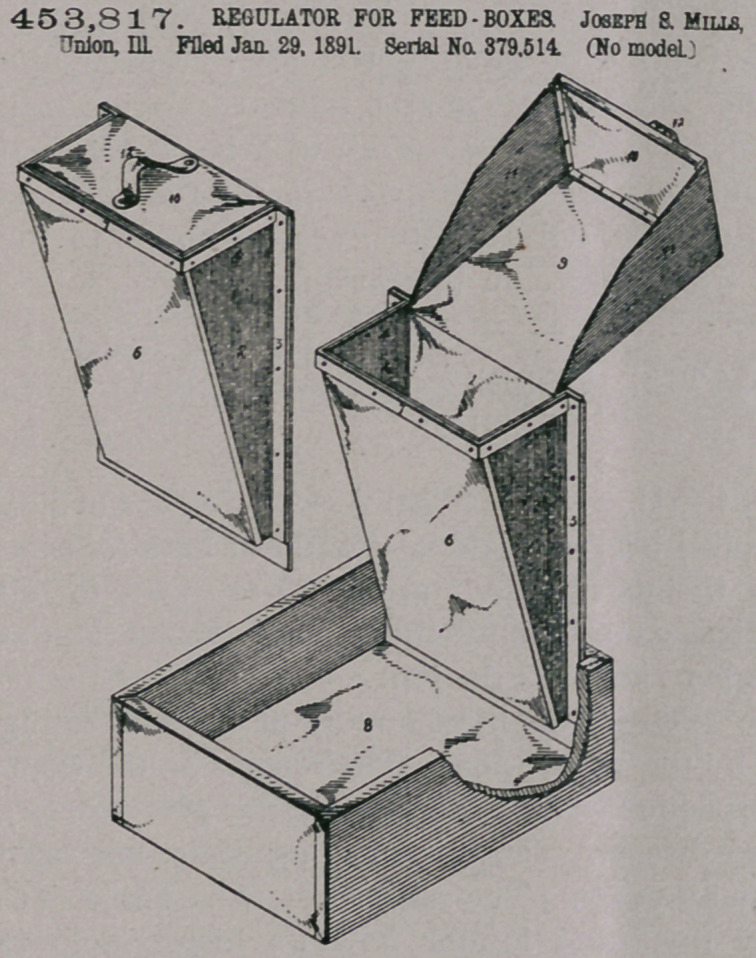


**Figure f15:**